# Rabies in the Dog in Its Relation to Rabies in Man

**Published:** 1886-04

**Authors:** Frank S. Billings


### THE JOURNAL

OF

### COMPARATIVE MEDICINE AND SURGERY.

VOL. VII. APRIL, 1886. No. 2.

### ORIGINAL COMMUNICATIONS.

### ART. VI.—RABIES IN THE DOG IN ITS RELATION TO RABIES IN MAN. WITH ESPECIAL REFERENCE TO ITS PREVENTION, AND TO PASTEUR'S SYSTEM OF PREVENTIVE INOCULATION.*

BY F. S. BILLINGS, D.V.S.

Rabies is the only word by which this disease should be designated in either man or animals. Hydrophobia is a misnomer with regard to the disease in either species. Neither dogs nor man when affected with this disease have any fear of water.

Dogs cross streams and endeavor to drink by dipping their mouths deep into vessels containing water. The rabied man longs and begs for it, but, with a shudder of frenzy, pushes it away, or demands it being at once taken from him because of the terrible sufferings which he knows will accompany any endeavor on his part to swallow it.

* The statistics, and most of the references in the following pages are taken from Mr. Fleming's "Rabies and Hydrophobia," which is still the best book of reference to be had upon this subject.

### WHAT IS RABIES?

Rabies is a contagious disease, *par excellence*, which apparently finds its origin in the canine (?) species only, and is imparted to other members of the same species, or to other warm-blooded animals by the bite of a rabid animal, most commonly a dog; the saliva being the vehicle of transmission, which holds the still unknown cause in suspension.

Like the greater number of our contagious diseases, in both man and animals, the primary origin of rabies is buried behind the myths and legends collected by the very earliest Hellenic and Egyptian writers. The most trustworthy author of this period, Aristotle, mentions the occurrence as a primary complaint in dogs, and its transmissibility to other mammalia, *with the exception of man.* The Greek and Roman physicians and authors that lived about this time did not make any such exception. We find many striking accounts of the terrible results of the bite of the mad dog in man, as well as many still more striking cures. It is not uninteresting to read that Pliny wrote: "When a person has been bitten by a mad dog he may be preserved from hydrophobia by applying the ashes of a dog's head to the wound," and, again, "Madness may be cured by eating a cock's brain." These passages indicate how old is the idea that the brain is largely the seat of the disease, but also that these early observers believed that the elements of rabid animals contained curative properties, as is still more evinced by the following passage: "There is beneath the tongue of the mad dog a certain slimy saliva which, taken in drink, is a preventive of hydrophobia." That this idea was not lost is evidenced by a quotation from an ancient MSS., or "Leech Book," written in the early part of the eleventh century. "A mad dog's head and liver, sodden, and given to be eaten to him who was torn, wonderfully healeth him."

### THE NATURE OF THE CAUSE IN RABIES.

It is unfortunate that all our English veterinary works advocate the "spontaneous generation" of contagious diseases: in this regard Mr. Fleming says of Rabies, "there are few now-a-days who are not convinced that it will occasionally appear without any certain assignable cause." * Dr. Hammond, † also supports the spontaneous idea in a way which is scarcely in harmony with the general character of his admirable work. He says. "It is very probable that the saliva of healthy animals is, under certain circumstances, capable of producing hydrophobia in man or other animals."

The most exact study of this question under the light of to-day cannot discover a particle of evidence in favor of any such hypothesis or assertion.

Another most serious assertion that runs through the writings of nearly all medical writers on contagio-infectious diseases is that their etiological moment is a "poison."

A poison is a destructive agent, capable of isolation by the principle and methods of chemistry, having none of the attributes of life; *i.e.*, it cannot increase its own quality or quantity by the multiplication of its own elements; if introduced into an animal organism a poison can only be depended upon to act when a certain definite quantity has been given: if not fatal, the action becomes less and less as the poison is eliminated from the system by the appropriate organs.

How different is the action of the infecting elements in all contagious diseases, so far as we are acquainted with them! They are vital organisms; they have all the properties of life in their infinitesimal bodies: they take up nourishment and give off effete material: they can reproduce themselves in their own kind: no certain quantity is necessary would we produce a given disease with them. The smallest number of active spores that we can take up on the almost invisible point of the finest needle will produce anthrax in an inoculated animal just as surely as the countless numbers necessary to produce the smallest perceptible mass. They continue to live and reproduce so long as the body offers the suitable nutrition: the excretory organs have no perceptible influence upon their action or numbers. The infectious principle in rabies

---

* The above was written in 1872. There is every reason to think that this statement is not in accordance with the author's present views, for, at about the same time he advocated similar ideas in regard to glanders, but has since given them up entirely, and joined the contagionists.

† "Diseases of the Nervous System, 1881."

must have these characteristics, it must be a micro-organism. It must be held in suspension, not in solution, in the saliva.*

In that most unjust and mistaken publication, "Louis Pasteur: His Life and Labors," by his son-in-law, and translated into English by Lady Claude Hamilton, with the endorsement of Mr. John Tyndall, one may read among numerous other misstatements the following in regard to the discovery of a specific microbe in rabies: "This microbe of the saliva is very easily cultivated in sterile infusions, and successive cultures can be made in the usual way." "The virulence continues." "Thuillier has had the patience to make, in this manner, eighty cultures in contact, with the air, and eighty cultures in a vacuum, * * * The eightieth culture killed as quickly as the first," (p. 293).

On page 299 of the same book we find these statements are flatly contradicted, as follows: "For ourselves, as long as the cultivation of the microbe outside of the organism has not been affected, and that hydrophobia has not been communicated by means of artificial cultures, we shall abstain from expressing a definite opinion on the subject."

M. Pasteur himself, as well as his very able assistants, MM. Roux and Wasserzug, assured me that they had been thus far unable to isolate, color, cultivate or prove the existence of a specific microbe by direct experiment with cultivated material, either from the nervous substance or saliva of rabid animals.

Practical experience, as well as the thousands of inoculations that have been made with the saliva of rabid dogs, from the very earliest dates of medical literature, have all sufficiently demonstrated that the contagious principle is contained in it.

It was the result of the methodical observation of an exact scientist that led Louis Pasteur to look upon the substance of the brain and spinal cord as the tissues of the body in which the infectious principle would be most likely to be found in a pure state. As already said, notwithstanding the most determined research at the hands of such a master of bacteria

* I recently read an article in favor of the poisonous nature of the saliva, in which it was asserted "that in all cases where rabies followed the bite of a dog, it would be found that the animal had a hollow fang like a snake connecting with the sublingual gland.

staining and isolation as M. Roux is known to be, still there has been no discovery of any specific microbe at Pasteur's laboratory. On the contrary, Fol, of Geneva, has recently published (Jan., 1886), advance notes of the discovery of a coccus in the substance of the medulla which he claims to have stained by Weigert's method of using hæmotoxlin for nerve substance, with some peculiar modifications. He also claims to have produced rabies in rabbits with pure cultivations of the same.

This last assertion is sufficient to throw doubt on these statements. In order to prove what he asserts, Fol should have used dogs, and have produced genuine rabies in a series of these animals, or else have carried his supposed rabbit rabies to dogs, and proved the correctness of what must at present be considered an hypothesis.

As said above, Pasteur has shown conclusively that the substance of the brain and cord contains the infectious principle in an active and available form. In Fol's experiments it is stated that he used the fluid pressed out of the healthy brain of a sheep as a cultivating medium. When I first read the passage quoted from the life of Pasteur as to the cultivation of a "specific microbe" from the saliva of rabid dogs, I was at once struck with the brilliancy of such isolation, but soon lost my enthusiasm through the ensuing contradiction. That these unknown elements, are suspended in the saliva is beyond all question, but every bacteriologist must be fully aware of the extreme difficulty of isolating specific bacterium, especially such a minute organism, perhaps a coccus from a medium so full of micro-organism as the saliva of a rabid dog. These elements must be in the saliva, however, and in it before it enters the mouth, which indicates several practical experiments to be completed in the future.

1st.—To place a salivary fistula on the parotid ducts of a large rabid dog, and thence lead into appropriately sterilized vessels.

2d.—To make inoculations upon other dogs with such material.

3d.—To make cultivations from the same.

4th.—To use the parotid and sub-lingual glands in the same manner.

5th.—To try feeding experiments upon healthy dogs.

(a) With bread soaked in the saliva of rabid ones.

(b) With glandular substances.

(c) With brain and cord material.

6th.—That this parotid secretion, which can be obtained in any desired amount by placing a fistula upon the morbid ducts of cattle, offers the most natural material for cultivating experiments. It can be used in its natural fluid form by exercising great care in dissecting out the ducts, and at once leading the cut end into properly sterilized and closing vessels.

It can be prepared as a solid medium by treating it as in the preparation of blood serum, or as a gelatinous preparation for plate cultures by first carefully precipitating the albumen by heat (the degree necessary must be watched so as not to overdo it), and then adding gelatine as in the ordinary method of preparing meat-infusion-gelatine.

It would also seem advisable to again repeat the experiments that have been made with the flesh, milk and other tissues of rabid animals. That people have eaten such material, that rabid bitches have retained their affection for their young and suckled them until no longer able to do so, and no evil effects resulted therefrom, has been quite frequently observed. The ancients were certainly Isopaths in this direction, for we find that they recommend that persons bitten by rabid dogs should eat the liver or some portion of the same. I remember seeing quite an intelligent dog fancier cut a bull-dog open alive, that had just bitten him, and taking the beating heart out and splitting it open, bind it warm on the parts bitten, "in order to prevent hydrophobia," as he told me.

### HYPOTHETIC CAUSES.

Among these may be mentioned *heat*, which finds its corroboration in the popular ideas connected with the so-called "dog days." No greater absurdity could exist. There is scarcely a doubt that rabies extended over Europe with the Roman armies, and thus became domesticated in the northern parts and Britain. Dogs have undoubtedly been the one great active element in its extension to other countries. When Mr. Fleming wrote—1872—it was not known in Australia, New Zealand, and various insulated portions of the world. It is not certain that it has ever extended to the Arctic regions. It has been reported that it did not exist in Constantinople, but Ahmed Effendi, Professor of Veterinary Medicine in the Military Academy at Constantinople, stated at the International Veterinary Congress at Vienna, 1865 : "That in the East—Turkey—the disease exists, etc." It is, however, singular that there is so little of it there when we consider that the semi-wild dogs of that city are the public scavengers, and are continually growling, snapping and fighting over their filthy plunder. It frequently occurs in Algiers and Mexico, and less so in South America.

Our recent experience in this country shows that if nothing else, we can have local attacks of rabiphobia in our people in winter, notwithstanding the evil repute of the hot months. Statistics do not seem to favor the warm months, as is shown by the following, with regard to dogs admitted as rabid or suspected at the Veterinary School, Lyons, France, during the year 1865 :

| | Rabid. | Suspected. | Became Rabid in Hospital. | Total Number admitted. |
|---|---|---|---|---|
| January, | 12 | 2 | 0 | 14 |
| February | 14 | 4 | 1 | 18 |
| March, | 6 | 11 | 0 | 17 |
| April, | 14 | 20 | 1 | 34 |
| May, | 12 | 14 | 1 | 26 |
| June, | 6 | 14 | 1 | 20 |
| July, | 2 | 8 | 2 | 10 |
| August, | 7 | 3 | 2 | 10 |
| September, | 1 | 3 | 0 | 4 |
| October, | 3 | 1 | 0 | 4 |
| November, | 0 | 0 | 0 | 0 |
| December, | 1 | 1 | 1 | 2 |

One of the absurd ideas which found advocates for many years was that, because there are so many more males than females, the former could not indulge their sexual passions sufficiently and hence got mad.

Neither want of water, ill-treatment, pampering, nor any one of the many things that have been asserted to play an etiological role in canine rabies have anything to do with it.

### RABIES DUE TO NATURAL INFECTION.

Limiting ourselves to the consideration of canine and human rabies, and in consideration of the late rabiphobia over our country, it may be well to introduce this subject by statistics.

In and around Paris there have been for years an average of about 150,000 dogs, about one-third of which are licensed. The number of actually rabid dogs admitted to the Veterinary School at Alfort, for a period of ten years, was:

| | |
|---|---|
| 1853, | 11 |
| 1854, | 3 |
| 1855, | 16 |
| 1856, | 20 |
| 1857, | 17 |
| 1858, | 19 |
| 1859, | 17 |
| 1860, | 20 |
| 1861, | 37 |
| 1862, | 32. |

19.1-5 dogs a year out of 150,000.

There are no statistics as to rabies in dogs in any part of the United States. The following, however, kindly furnished me by Professor Liautard of the American Veterinary College, are not without instructive interest. They include every dog that has died of rabies in that institution from the year 1874 to February 10, 1886.

| | | | | |
|---|---|---|---|---|
| Admitted, | November, | 1875, | 1 spotted dog, | died of rabies. |
| " | August, | 1876, | 1 terrier | " " |
| " | September, | " | 1 " | " " |
| " | October, | " | 1 spitz | " " |
| " | February, | 1879, | 1 pug | " " |
| " | March, | 1880, | 1 spotted | " " |
| " | September, | 1881, | 1 pointer | " " |
| " | " | " | 1 bull | " " |
| " | November, | " | 1 terrier | " " |
| " | April, | 1882, | 1 spitz | " " |

The mortality from rabies in human beings for a term of years in England is given in the Register General's Reports thus: "From the years 1851–1867, inclusive, the number of deaths from rabies to the million inhabitants was:"

| | | | | | | | | |
|---|---|---|---|---|---|---|---|---|
| 1851, | 1852, | 1853, | 1854, | 1855, | 1856, | 1857, | 1858, | 1859. |
| 1 | 0.8 | 0.6 | 0.9 | 0.1 | 0.3 | 0.2 | 0.1 | 0.2 |
| 1860, | 1861, | 1862, | 1863, | 1864. | 1865, | 1866, | 1867, | |
| 0.2 | 0.2 | 0.05 | 0.2 | 0.6 | 1. | 2. | 0.5. | |

Not one person in a million died of rabies in a year in a country where there has been known to be, at almost all times, much more canine rabies than we have ever had actual knowledge of in this country. At that ratio we do not have over 25 deaths from rabies in over 50,000,000 people in a year.

Is it not time that sensationalists were either confined or fined, for producing the nonsensical alarm we have lately gone through?

The following, taken from the Paris letter to the New York *Medical Record*, dated December 18th, 1885, bears especial relation to the questions at present under discussion :

"Rabies, which, during the period from 1880 to 1884, diminished in Paris in a notable manner, had become more prevalent at the end of last year, and has continued with scarcely any abatement to the present time. From the commencement of the present year twenty cases of death from the disease have already been registered in Paris, and this figure is the highest that has yet been attained. In consequence of the alarming increase of such a terrible malady, M. Leblanc proposed, at the meeting of the Academy of Medicine, last week, that the following measures should be carried out: 1. That the law of July, 21, 1881, which prescribed the declaration of all cases of hydrophobia, the destruction of all animals bitten, the wearing by dogs of a collar of a certain kind, and the taking to the pound those that are found straying. 2. To render general and uniform the organization of the service of epizootics, and to place this service under the direction of veterinarians. 3. To cancel the adjournment of the article of the law forbidding empirics to treat animals affected with contagious maladies. To these propositions, which were unanimously accepted by the Academy, M. Le Fort added another compelling owners of dogs to attach to the collar a small medal bearing a mark showing that the tax had been paid for the year. All dogs not wearing these articles will be treated as stray dogs. M. Dujardin-Beaumetz, in supporting the conclusions of M. Leblanc's report, pointed out the difficulties in carrying out these regulations. M. Dujardin-Beaumetz has been charged for the last five years to search for cases of rabies among the population of Paris. In his report he observed that, as the spontaneousness of rabies is universally denied, the governing bodies are bound to enforce such police regulations as may be necessary to prevent the spread of the disease. It would appear that these regulations are strictly enforced in Germany, where hydrophobia is by no means so common as it is in this country, and even in Great Britain. According to M. Dujardin-Beaumetz's report, there were in Paris, in 1881, 17 cases of death from hydrophobia; in 1882, thanks to the measures that had been taken, there were 11, and in 1883 there were only 6. In 1884, no case of the disease occurred in the first half-year; in the second half 3 cases were reported. In 1885, 20 cases have been registered to the present date."

The Secretary of the State Board of Health of Massachusetts reports that "there has not been one death a year from this cause in the last 30 years, and for the last 3 years not one."

Were it not for the terrible tortures to which our race is liable from the bite of a rabid dog, or were the disease strictly limited to that species of animal, rabies would not be a disease worth considering a moment in comparison with consumption that kills $\frac{2}{7}$ of mankind; pleuro-pneumonia that has cost us millions and threatens our entire cattle interests; hog cholera, that costs us at least $30,000,000 annually; trichinosis, that costs us one-half our pork export business, and during the month of January, 1886, is reported as having attacked 42 of our citizens; tuberculosis, that has almost ruined many cattle breeders, and is a constant source of danger to our cattle interests and to ourselves as consumers of beef and milk.

Experience has proven that the danger to the person bitten varies considerably with the condition of the part of the body attacked, whether it be covered or exposed. Bouley indicates the influence as follows: *

| | |
|---|---|
| Bites on the | face, fatal, 90 per cent. |
| " " | hands, fatal, 63 per cent. |
| " " | clad limbs fatal, 29 per per cent. |

In every contagious disease there is what is termed a period of incubation, *i. e.*, the time which elapses between the inception of the infectious elements and the period at which they first begin to manifest themselves by certain local or general disturbances.

This period of apparent inactivity is also known as that of latency. In rabies, the same is marked by an uncertainty and irregularity unknown in connection with any other contagious

* That the greatest circumspection is necessary in handling the cadaver of the rabid dog, or in making autopsies, or in the use of any materials which bave been in contact with such an animal, is evident to every one, but to make it more so, the following examples are appended. Youatt, relates of a man becoming rabid and dying from untying the knot in a rope with his teeth, by which a mad dog had been tied; he also tells of a woman that died of the disease from pressing the seams made in her dress after repairing rents torn in it by a mad dog. A. Mr. Lawrence mentions a case of a lady dying from rabies from letting a pet poodle lick her chin upon which there was a pimple. Fleming mentions numerous cases of a similar nature.—*Watson's Practice of Physics.*

disease. The reason is absolutely beyond our present means of elucidation, hence any attempt at theorizing over it would but show our own weakness. During the latent period the individual bitten shows no bad symptom. The wound heals remarkably well. In some cases pain has been reported for a few days, or even constitutional disturbances, but the latter, in human beings, are more probably the result of anxiety than of direct irritation in the trauma. This period of latency has also been called that of the localization of the infecting principle. The latter is at present an hypothesis, but we may yet be able to offer substantial evidence of its correctness. In no disease is this phenomenon so striking as in rabies. In none is there so much reason for assuming that there are some peculiar conditions, or perhaps, biological necessities, in connection with the etiological moment which under such localization is probable. I do not think it has anything whatever to do with the organism bitten, but, to restate it ; the localization of an infecting principle must be sought in the biological idiosyncrasies of the medium itself.

There are the most extreme divergencies in the reports of authors as to the extent of this period, more especially with regard to rabies in man, the time varying from six days* as a minimum to seven years.† The first extreme is so very rare as to be doubtful,and the second is deserving of still more skepticism ; in fact, reported cases of rabies in man of over a year's duration require the most exact revision. The following are some statistics as to the period of incubation in the dog.

| | | | | | |
|---|---|---|---|---|---|
| Lafosse | gives | the two | extremes, | from | 7 to 155 days. |
| Roell | " | " | " | " | 21 " 70 " |
| Youatt, | " | " | " | " | 17 " 210 " |
| Blaine, | " | " | " | " | 21 " 50 " |

But Blaine has also observed cases extend to 150 days.
Peuch gives reliable cases as occurring in 10, 15, 18, 22, 24, 26, 35, 88 and 95 days.
Renault gives a list with regard to 63 dogs.

Rabies developed in

| | | | | | | | |
|---|---|---|---|---|---|---|---|
| 1 dog | in from | 5 to 10 | days. | 7 dogs | in from | 45 to 50 | days. |
| 4 " | " | 10 " 15 | " | 2 " | " | 50 " 55 | " |
| 6 " | " | 15 " 20 | " | 2 " | " | 55 " 60 | " |
| 9 " | " | 25 " 30 | " | 4 " | " | 60 " 65 | " |
| 10 " | " | 30 " 35 | " | 1 " | " | 65 " 70 | " |
| 2 " | " | 35 " 40 | " | 4 " | " | 70 " 75 | " |
| 8 " | " | 40 " 45 | " | 2 " | " | 80 " 90 | " |
| | | | | 1 " | " | 100 " 118 | " |

---

* Hammond. † Fleming.

Saint Cyr gives a similar table which gives the minimum period of incubation as 16, and the maximum as 115 days, or an average of about 33 days. The statistics as regards man:

Romberg gives the shortest period of incubation as 15, and the longest as 270 days.
The average (England) period of incubation has been 40 days.

| | | | |
|---|---|---|---|
| Tardieu mentions | 26 | cases that occurred in less than | 30 " |
| " " | 93 | " " " " | 30 to 90 days. |
| " " | 19 | " " " " | 90 " 180 " |
| " " | 9 | " " " " | 180 " 365 " |

Of 86 undoubted cases.

| | | |
|---|---|---|
| 17 | occured in | 30 days. |
| 57 | " " | 30 to 90 days. |
| 6 | " " | 90 " 180 " |
| 6 | " " | 180 " 360 " |

It will be thus seen that the period of incubation in natural infection does not vary much in dogs or man, though we do not find the extreme limits mentioned with regard to the former that we do in the latter.

As is well known, there were six children and quite a number of dogs, six of which have been recognized and securely confined, bitten by a *supposed mad dog* on December 2, 1885, in the city of Newark, N. J. It is not necessary to recapitulate the nature of these wounds, a few of which were on the hands, but the majority were on the limbs, where the teeth had to penetrate through thick clothing. It will be remembered what intense excitement was created and sustained for some six weeks over the incident. That there were grounds for the same may be found in the facts that during the summer and fall a very reliable Newark physician had had two cases of fatal rabies in human beings ; the actions of the dog that bit these children were also so unnatural as to warrant the strongest suspicions as to its having been indeed rabid. In biting, ugly dogs generally attack, shake and tear another dog or object with some persistency, but the dog in question did none of this : the children and dogs bitten were in different parts of the city, but all in the path taken by the dog in his travels. That the animal was suffering from intense cerebral excitement there is no doubt : he snapped at the children, gave them a quick, sharp bite or two, and went on his way, only attacking objects directly in his path. He was finally killed and made away with without any satisfactory autopsy having been made.

As nearly three months have now elapsed since the children and dogs were bitten, there are the strongest reasons for assuming, as well as hoping, that the serious apprehensions entertained were groundless, especially as neither of the bitten dogs or the two children that were bitten, but not sent to Paris, have not shown a single constitutional disturbance since the event. Where so serious a case is found to be unwarranted we should learn from it to exert the greatest circumspection before giving support to the cry of "mad dog," and "hydrophobia," and thus create an unnecessary alarm which is always liable to be followed by untoward results in timid and sentimental people, especially if they should be accidentally bitten by any dog at such times. Such sensationalism, which was only too largely supported by many physicians, only to gain notoriety, is opposed to the spirit of exact science, and brings not only it, but the entire medical profession, into disrepute and distrust.

PREDISPOSING CAUSES AND PROTECTING INFLUENCES.

That the different species of animal life have a varying degree of receptivity, as well as different individuals of the same species, to the infectious principle of a given disease is well known.

With regard to rabies, however, there is much reason to think that the degree of non-receptivity, or constitutional immunity, is not nearly so great as has been generally assumed. Leaving all treatment of the wound out of the question, it is known that by no means 50 per cent. of the persons bitten by rabid and suspected dogs acquire the disease when we disregard the seat of the wound. Renault places this percentage at 33 per cent. Hertwig reports that of 137 dogs bitten in the streets of Berlin, in a given period, by rabid or suspected dogs, *but* 16 *became rabid.*

Renault claims that one-fourth of the animals directly inoculated with saliva from rabid dogs, escaped the disease.

Hertwig received positive results in 14 out of 59 dogs—23.7 per cent. treated in the same manner. In some cases he found several inoculations necessary, and his celebrated bull-mastiff was resistent to all inoculations for a period of three years, although seven other dogs died o frabies from the same material.

A general review of such inexact statistics as exist will show that about two-thirds of the individuals bitten by rabid or suspected dogs never acquire the disease, while by intentional inoculation, as said above, about one-fourth escape. It must be remembered that these inoculations, whether natural or artificial, have all been made with the saliva : also that there are innumerable circumstances which tend to mitigate against any constancy in the virulence of this material. Pasteur has, however, shown us that in the brain and spinal cord of rabid dogs we have a material which, on being artificially introduced into the sub-meningel space of the brain in healthy dogs has an unfailing constancy of action. The same is true of rabbits and guinea-pigs. On the other hand, that there is a special idiosyncratic, or partial immunity (even for such constantly active material as above-mentioned) in some of the mammalia, has been shown by Pasteur, to exist in monkeys. Other animals have not been tried to any great extent.

### DURATION OF RABIES.

In 131 cases of genuine rabies the average duration of the disease was five days : the shortest period was two days in the case of seven of these dogs ; seven days in the case of two ; nine days in two others, and thirteen days in one. In man, the shortest time reported is *one* day ; the longest, twenty days. In 234 cases the average duration of the malady was four days.

### PATHOLOGICAL ANATOMY IN THE DOG.

As in the majority of diseases which essentially complicate the nervous system the macroscopical pathological anatomy is very unsatisfactory. The microscopical is still more so, the specific microbe has not yet been discovered ; nor have any lesions been described which can be depended upon as pathognomonic to this disease.

A fact, probably more or less unknown to the majority of pathologists that have only worked upon lesions in man, is that the brain and cord in the healthy dogs are invariably more hyperæmic and reddish than in man, and also that the cross-section of these organs reveals a peculiar glance and transparency which in man would lead to the suspicion of a slight degree of hyperæmia and œdema.

In rabies, these conditions are ex-acerbated. Effusion into the cavities of the brain and cord, and in the meshes of the lepto-mervieux is by no means rare. That the lungs, liver, kidneys and intestines should be more or less hyperæmic, with ecchymotic conditions is not surprising.

A phenomenon which requires the greatest circumspection in expressing a conclusion as to the existence of rabies in a given case is the presence of foreign material in the stomach of a dog. The mere presence of straw, grass, horse manure, shreds of carpet, or chips in the stomach is not sufficient to warrant the diagnosis of rabies. When united with a very suspicious history; when the mucosa of the digestive tract, from the mouth to the anus, is intensely congested, and the seat of more or less tumefaction, with hæmorrhagic centers dispersed through it; when the fauces are swollen, parched and covered with a viscid adhesive mucous; when the stomach is intensely swollen; when there are extensive hæmorrhages into its substance, and the stomach contains a coffee-colored, hæmorrhagic mass in connection with all sorts of abnormal material then, and only then, can we begin to think of rabies with certainty.

The mucosa of the respiratory tract will generally be found in a similar condition. Lesions of the tongue and lips are to be looked upon with the greatest suspicion. The bladder is generally empty. It is highly important that this whole subject be again gone over, and subjected to all the new methods of pathological investigation.

PROPHYLAXIS, PASTEUR'S METHOD OF PREVENTIVE INOCULATION.

A critical study of the work of Louis Pasteur teaches us a lesson which must be learned in this country before we can ever hope to see original research receive support and become as it should be, a blessing and credit to our people. As with the completion of every great scientific work, we shall find that no one man has ever completed all the work upon which they depend.

First.—Pasteur had never been successful without the monetary support of his government.

Second.—Science develops by steps. Pasteur has brought to a certain degree of completion five tasks in his life that will remain monuments to his genius. He has been over forty years devotedly working at his mission. The managers of a certain laboratory erected in New York, and said to be devoted to original research (?), lately announced that within the next six months four original papers will appear which will represent original work done in Cargnegie Laboratory.

Compare such wonderful abilities with the forty years' devotion of Pasteur, with the twenty years' work of Robert Koch, which has resulted in two original works!

How insignificant such men appear in comparison with our American products. Even the world's mightiest Virchow must look upon us with envy, and Darwin rest uneasy in his grave! The dust of Aristotle must even seek to resume its original form again before such soul-inspiring abilities as we have here in New York.

What is original work?

Such as moves the wheel of progress one cog forwards, or more, so that it remains and does not rotate back again.

As already mentioned, the author of the "Life of Pasteur" almost entirely overlooks the work of others in the same fields of research. When reading of his investigations in connection with anthrax one is almost led to think that Pasteur had done about almost all, and no mention is made of the original discoverers of bacillus anthracis, his countryman Dovaine, or the Dorpat professor, Brauel. The wonderful biological work of the greatest living master in bacterial cultivation, Koch, in connection with this bacterium is entirely overlooked, and a polemic entered into against him because he had not accepted Pasteur's inoculation theories and experiments as conclusive. His great contemporaries in the study of and experimentation with preventive vaccines, those eminent French veterinarians, Chauveau, Cornevin and Toussaint, find no mention whatever. Yet it is a question if it has not been as brilliant and valuable to agriculture as the more sensational rabination of Pasteur will prove to man.

The fact that different species of animals possess a different degree of receptivity for the specific germs of the same disease, or better, the virus of a given disease, is much older than Pasteur's works on these subjects. Again, it is a mistake to attribute to Pasteur the discovery of the principle of the attenuation of such a virus by being passed through certain numbers of a given species of animals.

Renault anticipated him in asserting "that the rabaic virus of the carnivora, though potent in producing the disease in herbivora, lost this potency in them, and hence the non-virulence of their saliva."

The experiments of Prof. Rey, of Lyons, show that there is some foundation for the belief that repeated transmissions attenuate the power of the infecting element. By successive inoculations he found that rabid virus from a single primitive source appeared to act with much less promptitude on the last than in the first inoculated animal, and that in sheep its action ceased after its fifth removal or generation. These statements do not in the least detract from the character of the work done, or the reputation of Louis Pasteur. They only show the slow path by which scientific work is done. The world does not take sufficient cognizance of the work of the individual discoverers of facts—the sappers and miners of science—who have prepared the way and made possible the discovery, or better, the application of great general principles by such men as Virchow, Darwin or Pasteur.

It is a singular coincidence that the two greatest and most sensational discoveries to which the salvation of human life from accidental injuries is due have been made by Frenchmen. Some great French artist should attempt to re-mortalize the glorious painting of Ambrose Paré introducing the ligature on the field of battle, thus saving millions of lives, and other millions from the tortures of the seathing iron and scalding oil; on the opposite side of the picture he should portray another immortal, and picture some fond mother blessing that grand, quiet, self-devoted man, Louis Pasteur, for saving her child's life from a group of maddened, demoniacal dogs, that cower before the master's glance.

It has been already mentioned that to Pasteur is due the discovery of the valuable fact that the brain and spinal cord of rabid animals contained the virulent elements in a most active and thoroughly reliable form for experimentation. Having arrived at this hypothesis from the critical study of the phenomena of the disease in rabid dogs, the next thing to do was to prove its correctness by actual experiment.

The dog, itself, is the only really reliable animal upon which we can depend to test any such material. Pasteur next resolved to see what would be the effect of bringing some of this brain material from rabid dogs in direct contact with the brain of healthy ones. The result was the establishment of the actual period of infection in dogs in this disease. Numerous experiments prove that in about fifteen days rabid phenomena could be depended upon appearing. This great result is of great practical importance as a trustworthy means of diagnosis in case of suspicion of rabies in a dog, especially if human beings have been bitten.

If the dog has not been killed, a week's close and secure confinement will satisfy the question as to rabies, or not; but when it is impossible to catch and confine such a dog, and it has been killed, this method of intra-cranial inoculation can be depended upon to give satisfactory results.

The method of inoculation is simply that of ordinary trepanning, but the instrument should not have a diameter larger than the end of an ordinary lead pencil. Great care must be taken not to penetrate or injure the dura mater. In his first experiments, Pasteur used a piece of the infected brain substance simply, but subsequent experiences have shown that the material can be better depended upon when held in suspension in a fluid medium. An ordinary hypodermic syringe, with a curved needle is used to introduce the virus. A quantity equal to two divisions of the piston-rod is sufficient.

A virus is any material upon which specific infectious elements are bound: it may be fluid or solid, but is not the specific material itself, as is too frequently asserted. The animal must be chloroformed. The operation is terminated by suturing the skin in the ordinary manner. All antiseptic precautions must be rigidly observed.

The inoculation experiments were continued, and on account of their zöological connection with man, it was but natural that Pasteur should desire to test the results of such experiments upon monkeys. As previously mentioned, the fact was already well known, and had been well demonstrated in connection with some other diseases, that a specific virulent material may become ameliorated by being passed through a succession of animals of a given species; while in another species of animals the same material had been shown to have a steadily increasing force until a given limit has been reached. The number of animals necessary to produce either of these effects with a given virus has always been found to be approximately the same. In monkeys, Pasteur received the first mentioned result. He must have been, indeed, surprised to find that these creatures, so nearly related to man, should have, in contradistinction to the latter, a natural predispositional immunity against the action of the most active virus taken from mad dogs. It had some effect, but none of the terrible phenomena which characterize the disease in man, were observed. On passing this same cerebral material (originally taken from a mad dog and proved by correction experiment on other dogs) through a very short series of monkeys, it was found that it had ceased to have any visible action, which condition was also reproved on dogs. Hence the brain or spinal cord of a rabid dog will not produce characteristic rabies in monkeys, and if passed through a very small number of such animals the same substance, when taken from them, has no action whatever when re-tested upon dogs by the same procedure.

Whether such an apparently non-virulent material from monkeys will again acquire its original canine virulence on being again passed through a series of dogs has not been decided so far as one can judge from the very brief and incomplete publications of M. Pasteur in the "Comptes Rendus," nor was anything of the kind related to me while in Paris.

From his experience in the study of "Rouget," Pasteur was warranted in assuming that he had discovered in monkeys a medium by which he could provide himself with the material for a *primary preventive vaccine.* Unfortunately, monkeys are unsuitable, on account of their scarcity and consequent cost. It would be interesting to know if this constitutional indifference to rabies is shared in by the different race of monkeys, and how it varies in them.

The rabbit has been selected by experimentalists as the best suited of all animals for vicarious experimental sacrifice. In this way it has served the needs of science, and been made to contribute to our exact knowledge of biological processes in health and disease than all other members of the animal kingdom together. No man has probably made more use of this animal than Louis Pasteur, and it was but natural that he should have had recourse to them in his further researches for a preventive vaccine against rabies.

Although in that book on his life which has been so frequently alluded to, it is said that "the grand problem in regard to hydrophobia is, not so much the isolation of the microbe as the finding of a means of preventing this frightful disease." Still, every one who has worked in this field of research, or who is at all acquainted with the methods and aims of Pasteur, knows that it is to the solution of this problem, and the discovery, isolation and cultivation of this specific microbe, with the sequential production of a virus from it, that the great French master looks for the grand conclusion which can only make his work, in this direction complete, and by which alone a thoroughly practical virus can be obtained which will be adapted to universal application.

He inoculated these rabbits in the sub-meningeal space of the brain, exactly as he had his monkeys and dogs. The primary inoculations gave exactly the same results as had been obtained in dogs. The brain, or cord material taken from rabid dogs produced rabbit rabies in about fifteen days. Rabbit rabies is not, however, characterized by the furious phenomenon which is common to that disease in dogs and man ; it is a form of progressive paralysis : there is no excitement or tendency to bite, hence these animals should never be used for that purpose.

The brain substance from these primarily-inoculated rabbits when re-inoculated on dogs, produced rabies again in them in about fifteen days, thus demonstrating the constancy of the material in its active characteristics.

On proceeding with these inoculations through a series of rabbits, it was found that this material gradually augmented in virulence, until after having been passed through a series of from twenty to twenty-five rabbits in a successive manner, it demonstrated an ability to cause the characteristic phenomena of rabbit rabies in about eight days, and finally, after having been passed through another series of rabbits, a maximum of virulence was attained in such cerebral material that it caused rabies in rabbits in seven days, and when passed from rabbit to rabbit, retained this constancy of character.

It has been, as yet, impossible to find a method by which a material could be produced that would shorten the period of inoculation less than the mentioned seven days. Such a material from rabbits will cause rabies in healthy dogs when inoculated in the same manner in from eight to ten days, which sufficiently demonstrates its augmented virulence as compared with the material primarily taken from rabid dogs that required fifteen days to produce the same effect.

To recapitulate :

1. Brain material from a rabid dog inoculated into a healthy dog's brain, has a constant period of infection of about fifteen days.

2. The same material, when inoculated into any number of individual rabbits, has also a constant period of infection of about fifteen days.

3. When the material from one of these rabbits is carried through a succession of rabbits, it finally acquires a constancy of action that will cause rabies in rabbits in seven days.

4. This last material, inoculated into dog's brains, causes canine rabies in eight to ten days.

It took three years to discover and satisfactorily confirm the above facts.

In December, 1885, a child that had been bitten thirty-five days previously by a mad dog, was treated by M. Pasteur, not without objection, however. Before its removal from Paris the child died of rabies. The opponents of Pasteur were filled with joy. They asserted that "his treatment was not only futile, but dangerous; the child may have died of induced rabies, and not from the dog bite."

Pasteur was not at all disconcerted. He had an autopsy made upon the child, and taking some of its brains, inoculated several rabbits (I saw them). As the symptoms which are characteristic of this disease did not appear until the seventeenth day, and as the child had latterly been inoculated with a virus from rabbit material that had been proved to possess the seven-day virulence, Pasteur defiantly asserted "the child came too late : it died of canine rabies."

Can rabies be differentiated from pseudo-hydrophobia in man by Pasteur's method?

Yes, if fatal.

The following case illustrates this point:* "Dr. Dujardin-Beaumetz made another report to the Council of Hygiene of the Seine to the effect that a case of death from hydrophobia recently occurred at the Hôtel Dieu, and that he had reason to believe that the disease came on spontaneously, as the body bore no marks of having been bitten. The patient was, however, epileptic, and this circumstance would tend to confirm Dr. Dujardin-Beaumetz's conclusion, though the patient denied having ever been bitten. Nevertheless, experiments performed by M. Pasteur on two rabbits which he inoculated with fragments of the medulla oblongata of the patient proved that the latter died from hydrophobia."

Having thus discovered a method by which a most virulent material could be obtained in any desired quantity and be available as a "secondary," or test vaccine, it remained for Pasteur to find some method of attenuating it so as to place in his hands a very weak material which could be used as a primary, as well as material which could be graded at will to assume any intermediate conditions.

In his researches after a virus to be used against anthrax he had discovered that while the bacilli of that disease developed spores at a temperature of 37° C., that when subjected to a constant temperature of 43° C., in a thermostat, the cultivations of these bacilli no more developed spores in bouillon, and that, with each day's exposure to this temperature, the cultivations gradually lost their virulence until it almost entirely, if not wholly, disappeared, when such constant exposures had extended over fourteen days.

Another wonderful fact was also discovered, viz., a bouillon cultivation of this character always retains approximately the same degree of virulence, according to the period of exposure to this temperature. Hence, a bouillon thus treated fourteen days constituted a *primary*, or almost non-virulent vaccine; one that had been exposed ten days is more virulent, and so on to the first, or strongest bouillon virus.

---

* Paris Letter to *Med. Record*, Dec. 18, 1885.

Experiments on animals have shown him, and been retested by others, that if an animal is serially treated by inoculation with this anthrax virus, beginning with the so-called primary, or fourteen-day bouillon, and after the lapse of ten days, succeeding with the stronger, that in the course of a few weeks a constitutional condition is produced in the animal—sheep especially, which renders it immune against the action of the strongest, or natural, virus.

Subsequent observations have shown that this produced immunity can be depended upon to last about a year. The next question was how to produce a similarly reliable virus from the brain substance of rabid rabbits. Had the "grand problem" of the discovery of the microbe been successful, it is very probable that the method would have been perfected much sooner, and in a far less inexpensive manner, both in money and animal life, than is possible under the present one.

PASTEUR'S METHOD FOR THE PREPARATION OF HIS ANTI-RABIES VIRUS.

The previously-considered studies upon bacillus anthracis indicated a probable method, but the cerebral, or cord material, had to be used instead of pure cultivations of a specific bacterium.

It was determined to try the effects of heat and cold upon such material, and, as is evident, the moisture in it might have some deleterious effect, a method had also to be found for abstracting the latter from the nervous substance as well as from the air in the vessels to be used.

Specimen jars, holding a pint of liquid, with hooks on the inside of the covers, are just the thing for this purpose. They must be cleanly washed and thoroughly sterilized; in the bottom of each jar must be placed enough of the driest caustic potash, in lumps, to fill the jar about one-third. The cover must be air-tight. The brain substance, not being suitable for suspension, and the spinal cord having been found to possess the same degree of activity, the medulla oblongata presenting itself as the most accessible portion of the cord, was taken for this purpose. This must be taken out with the utmost care, and with the use of the most carefully sterilized instruments. It must not come in contact with blood or extraneous matter. It is then to be fastened by one end to a previously sterilized thread, the other end of the thread to be attached to the inside of the glass cover. The procedure must be done as quickly as possible. The jars are to be then placed in a warm room where the temperature can be constantly held at 20° C. The result of the exposure to this hot dry air of these pieces of medulla has shown that allowing for the effects of a slight variation in thickness, they lose in virulency with each day's exposure. At the expiration of fourteen days they are no longer able to produce an marked effects when introduced into the sub-meningeal space of either rabbits or dogs. When the bottles containing the cords are placed in a room with a temperature just above freezing, the cords retain their virulency a long time.

Pieces of such cords, about one-fourth of an inch in length, are to be rubbed up in a sterilized conical glass with sterilized bouillon, or distilled water. About a tablespoonful of the fluid must be added, a few drops at a time. The glass rod used for the purpose must be clean, and thoroughly sterilized. The material must be prepared fresh each time, and no cord allowed to stand over to be used again. If not used at once, the glasses containing the virus must be carefully covered. It is not to be filtered.

As with anthrax virus, so with this material. Pasteur found that it had a certain constancy of virulence, according to the time of exposure, that could be depended upon within very narrow limits, *i.e.*, a cord that had been thus treated for fourteen consecutive days had little or no pathogenetic action ; one that had been thus treated for twelve days had more ; one of eight days still more, and so on, increasing in virulence, until the fresh, or extra-virulent cord was arrived at.

The next procedure was to test this material upon a given number of dogs by beginning with a virus made from a fourteen-day cord, and each day inoculating a material made from a cord that had been exposed to the hot dry air one day less, until a material was inoculated that would cause rabies in a rabbit in seven days, and in a dog in from eight to ten. This was done in hundreds of cases.

### CAN MAN BE SAVED?

The question had no sooner suggested itself than the victim to be saved was, as it were, providentially supplied in the Alsatian boy, Joseph Meister, aged nine years, whose death Pasteur judged to be inevitable, from the nature of his wounds, and the decidedly mad character of the dog that bit him. The inoculation of this boy began on the evening of the 6th of July, 1885 (sixty hours after he had been bitten) with the injection of one-half of a hypodermic syringe full of the artificial virus, beginning with that from a cord that had been treated fourteen days.

The following summary of this boy's treatment is taken from the *Comptes Rendus*, of October, 1885:

| Date. | Time of inoculation. | Date upon which rabbit was inoculated from which the spinal cord was taken. | No. of days the cord was dried after removal. |
|---|---|---|---|
| July 7 | 9 A.M. | June 23, 1885 | 14 |
| " 7 | 6 P.M. | " 25, " | 12 |
| " 8 | 9 A.M. | " 27. " | 11 |
| " 8 | 6 P.M. | " 29, " | 9 |
| " 9 | 11 A.M. | July 1, " | 8 |
| " 10 | 11 " | " 3, " | 6 |
| " 11 | 11 " | " 5, " | 7 |
| " 12 | 11 " | " 7, " | 5 |
| " 13 | 11 " | " 9, " | 4 |
| " 14 | 11 " | " 11, " | 3 |
| " 15 | 11 " | " 13, " | 2 |
| " 16 | 11 " | " 15, " | 1 |

At the same time the child was inoculated, a rabbit was inoculated with exactly the same material. This is done with the virus used on every person inoculated by M. Pasteur in order to keep the experiments under the most exact control. The result is thus reported by M. Pasteur:

"The medullas or cords which were removed from rabbits and inoculated on the child July 11, 12, 14, 15 and 16, respectively —that is, which had been desiccated seven, five, four, three, two and one day—all gave positive results when re-inoculated upon rabbits in a degree corresponding to the time they had been dried—*i.e.*, the freshest cords caused the appearance of rabies in the rabbits in seven and eight days, while the others gave results later and the driest gave none."

The fresh cords having therefore caused the outbreak of rabies in the regular time, which the most virulent cords from rabbits had previously done, and the same not having caused any disturbance whatever to appear in the boy Joseph Meister, Pasteur concludes "that the boy had not only escaped a future outbreak of canine rabies, but also that the systematic inoculation had so prepared the elements of his body that they were enabled to resist the action of still more virulent rabbit rabies.

In this way, Pasteur has now treated some 150 human beings, and every one of them has received, at the end of the treatment, a virus that would cause rabies in from eight to ten days.

Human beings are inoculated in the abdominal region, care being taken not to repeat the operation on the same side of the body on any consecutive days. Care is also taken not to completely empty the syringe, as the material is not filtered; an amount representing about one division line on the piston is always left in the syringe. Persons over fifteen years old receive a syringe-full; those under, about half a one; the operation is performed from ten to twelve times in the manner above depicted; no inflammatory or constitutional symptoms have ever resulted. Thirty days subsequent to the bite of a rabid dog is about the period that Pasteur considers his treatment can be depended upon.

### PASTEUR'S THEORY AS TO THE ACTION OF HIS VIRUS.

In explaining his views upon this subject to a correspondent of the London *Times*, Pasteur said "that the virus acted very slowly, and, while he was making the body refractory to it by repeated inoculations, the virus deposited by the bite localized itself in the region of the wound. Wherever this region, that virus becomes digested during the year and a half which he has found, by experiment, the inoculation lasts, and will no longer exist in the body. As the propagation of the virus, which has always an ascending tendency and directs itself to the brain, takes place so slowly that the minimum of the total inoculation, with it, is thirty days, the whole question consists in inoculating the patient soon enough to prevent the propagation of the virus through the wound from spreading." Again he says,* "drying the marrows in contact with the air is an effect of impoverishment in the quantity of the virus contained in them, and not an effect of its impoverishment in virulence."

I cannot agree with any of the above conclusions.

1st.—A virus is not the *cause* of a disease. It is, as already said, a something upon which the true, essential cause is bound.

2d.—The last quotation is simply a mere playing with words. If the pieces of the spinal cord have lost in activity they have also lost in virulence. The question is not one of quantity (of the virus), but of change (in active quality) which the specific germs have suffered from the treatment the cords have been subjected to. The action of the same is upon these germs not upon the cord in which they are lodged, as such.

3d.—The hypothesis that the immunity of the individua, bitten is caused by the "digestion" of the virus (?) at the locus tromaticus, or that the inoculation acts specifically and directly, so as to prevent the propagation of the virus through the wound, and spreading, will not hold good, theoretically.

The virus in this case is the saliva of a rabid dog; the infectious elements, the unknown, yet specific microbe. A virus can never propagate; what it holds in suspension, or what it plants in the wound, can. To my mind, this prolification is very likely to occur, but to what degree I am unable to say.

The immunity must be caused by the cell-elements of the bitten organism becoming gradually accustomed to the action, or influence, of these weakened elements as introduced in the inoculated material until, like a person living in a yellow-fever region, they become so acclimatized, as it were, that these same elements when introduced in the highest activity can produce no influence upon the organism. They may, or may not be, a prolification, and generalization of the germs at and from the locus tromaticus, but their action has been negatived, they may be carried over the system, but finding no lodging-place, they pass by on the other side, as it were; they die, or undergo disintegration, and are removed by the appropriate ways.

---

* "Popular Science," No. III., 1886, pp. 293, 295.

### OTHER METHODS OF PREVENTION.

*Cauterization.*—Strong carbolic acid is the best material for this purpose when it can be had. It is more thorough and less painful than fuming nitric acid or the hot iron, and makes a wound that heals easily and quickly.

*Legal Restrictions.*—High license fees, and the most rigid collection of the same, with the absolute slaughter of all unlicensed dogs is one of the best preventive measures against canine rabies. A special breeder's license should be issued for bitches, to be held for that purpose only. All other bitches, no matter by whom owned, should be refused a license, and at once killed, *unless spayed*, by a person authorized by law to perform the same, and to issue a certificate therefor. These measures will lead to diminishing the number of cur-dogs to the lowest possible limit.

It is possible to make Pasteur's method of inoculation so practical that every licensed dog in the country can be inoculated. The States must supply laboratories and the means for doing it.

The following regulations should be adopted and made obligatory in each State : *

Dogs in which signs of rabies appear, or which indicate suspicious phenomena, must either be at once killed by the owner, or those having them in charge, or be placed in secure confinement until the police have decided what is to be done with them.

When a human being or an animal has been bitten by a rabid or suspected dog, or been in any relation with such, so as to render infection probable, the suspected dog is not to be killed, but must be safely secured, when such can be done without further danger, until the police decide upon the course to take.

The transport of a dog in which rabies is suspected, for the purpose of confinement, must either take place in a closed vehicle, or the dog must be securely muzzled and led by two strong chains between two conductors.

---

* From the "Laws for the Suppression of Contagious Animal Diseases in Germany."

It is the duty of the local police to immediately cause the examination of a dog suspected of rabies by an official veterinarian, or when time would be lost in waiting for the attendance of the same, by an approved veterinarian. If there is a well-founded suspicion that the suspected dog has been in relation with a rabid or suspected dog, said dog must be at once securely confined and watched for a period of six days, if the examination of the veterinarian does not at once confirm the suspicion. If the dog lives over this time, and no suspicious phenomena appear, it is to be discharged.

If a dog suspected of rabies be at once killed, or if it dies during confinement, it is the duty of the police to order a *post-mortem* examination by an official veterinarian, if there has been any probability of men or animals having been bitten by said dog, or if said dog has been roaming over the country.

If the existence of the disease is confirmed, it is the duty of the police to make it known to the public in the usual way. Dogs in which rabies has been diagnosed must be at once killed. It is further the duty of the police to at once kill all dogs which have been bitten by a rabid dog, as well as all such as have been in relation with said dog, so as to render the further extension of the disease impossible.

When a rabid or suspected dog is known to be freely running over a section of country, it is the duty of the police to order the secure confinement of all other dogs in the district. All places in which the rabid or suspected dog has been seen are to be looked upon as dangerous, as well as a territory extending four kilometers beyond them. If the suspicion is unfounded, the confined dogs are to be again freed from restriction; if, on the contrary, the suspicion is confirmed, these restrictions must be extended over a period of at least three months. All dogs found running free, in opposition to said restrictions, must be at once killed by the police.

